# Does Obesity and Procedure Type Increase the Risk of In-Hospital Mortality in Laparoscopic Hysterectomy: A Report From the United States Hospitals

**DOI:** 10.7759/cureus.9332

**Published:** 2020-07-22

**Authors:** Chris A Robert, Mary P Robert, Rikinkumar S Patel

**Affiliations:** 1 Obstetrics & Gynecology, Sunrise Hospital, Pune, IND; 2 Obstetrics & Gynecology, LLH Hospital, Abu Dhabi, ARE; 3 Psychiatry, Griffin Memorial Hospital, Norman, USA

**Keywords:** laparoscopic assisted vaginal hysterectomy, laparoscopic hysterectomy, comorbid obesity, nationwide inpatient sample, in-hospital mortality, total laparoscopic hysterectomy

## Abstract

Objectives

To assess the differences in demographics and laparoscopic hysterectomy type by comorbid obesity and to assess the risk of in-hospital mortality due to obesity and other comorbidities.

Methods

We conducted a cross-sectional study using the Nationwide Inpatient Sample (NIS, 2012-2014), and included 119,890 adult females undergoing total laparoscopic hysterectomy (TLH), laparoscopic-assisted vaginal hysterectomy (LAVH), and laparoscopic supracervical hysterectomy (LSH). We used a logistic regression model adjusted for confounders to assess the odds ratio (OR) of obesity on mortality in study inpatients.

Results

The majority of the inpatients were middle-age 36-50 years (83.1%) and White (67.7%). Comorbidities were seen in a higher proportion of obesity cohort with most prevalent being hypertension (53.6%) and diabetes (23.9%), followed by depression and hypothyroidism (15.8% and 15.4%, respectively). Inpatients with comorbid obesity had 4.6 times (95% CI 2.79-7.69) higher odds for in-hospital mortality compared to non-obesity cohort. There was statistically no significant association between type of laparoscopic hysterectomy and in-hospital mortality.

Conclusion

Analysis of national-level data shows that obese patients have a higher risk of in-hospital mortality by 364% compared to non-obese patients. There was no significant association between the laparoscopy procedure type and in-hospital mortality. More studies should focus on improving hospital outcomes and quality of life post-surgery in obese patients.

## Introduction

Hysterectomies are the second most commonly performed procedure in women of reproductive age, and nearly 600,000 hysterectomies are performed annually. About 20 million women in the United States (US) have had a hysterectomy, and 52% of all hysterectomies were performed in women under 44 years of age [1]. Laparoscopic hysterectomy was majorly performed for benign indications (83.2%), with total laparoscopic hysterectomy (TLH) accounting for 48.3% followed by laparoscopic-assisted vaginal hysterectomy (LAVH, 37.3%), and laparoscopic supracervical hysterectomy (LSH, 14.4%) [2]. TLH is the preferred mode of hysterectomy as it affords various advantages, including low postoperative pain, less intraoperative and perioperative blood loss, shorter hospital stay, short convalescence period, and reduced infection rates compared with conventional abdominal hysterectomy [3-5].

According to the Centers for Disease Control and Prevention (CDC), the prevalence of obesity increased from 30.5% to 42.4% from the years 1999-2000 through 2017-2018. The prevalence was highest among middle-aged women (44.8%) and middle- and lowest-income groups. The medical cost of obesity has been estimated at $147 billion in the United States, with the average medical cost $1,429 higher in obese [6].

Obesity is often associated with comorbidities like hypertension, diabetes mellitus, and coronary disease and studies show a higher incidence of perioperative deep vein thrombosis, pulmonary embolism, and surgical site infection [7,8]. However, studies examining the role of obesity in laparoscopic gynecological surgeries have discrepant results with some showing increased risk of complications and conversions to laparotomy, and others showing no difference in between the different weight groups [9,10].

In our study, we aim to assess the differences in demographics and laparoscopic hysterectomy type by the presence of comorbid obesity, and next is to assess the risk of in-hospital mortality due to obesity and other comorbidities.

## Materials and methods

Data source

We conducted a cross-section data analysis using the healthcare cost and utilization project’s (HCUP) Nationwide Inpatient Sample (NIS) data from 2012 to 2014. The NIS provides patient records from about 4,400 non-federal hospitals and covers 45 states in the US. Diagnostic and procedure information in the NIS is identified using the International Classification of Diseases, ninth edition (ICD-9) codes, and Clinical Classification Software (CCS) codes [11].

Inclusion criteria and outcome variables

We included adult patients (age 18 to 50 years) with a primary procedure of laparoscopic hysterectomy, that is, TLH (ICD-9 codes: 68.41 or 68.61), LAVH (ICD-9 codes 68.51or 68.71), and LSH (ICD-9 code: 68.31). This sample was further grouped by comorbid discharge diagnosis of obesity using the ICD-9 codes 278.0, 278.00, 278.01, 278.03, 649.10-649.14, 793.91, V85.30-V85.39, V85.41-V85.45 or V85.54.

Demographic variables studied included age (18-35 and 36-50 years), and race (white, black, Hispanic, and Asian/Pacific Islanders, Native American, and others). The comorbid diagnosis for depression, diabetes, hypertension, and hypothyroidism were identified using ICD-9 diagnosis codes. We measured the in-hospital mortality between obesity and non-obesity cohorts and in the NIS, we in-hospital mortality is reported as all-cause [12].

Statistical analysis

We used cross-tabulation and descriptive statistics to discern the demographic and comorbidities differences in the sample population undergoing laparoscopic hysterectomy by comorbid obesity. We used another model of cross-tabulation and descriptive statistics to evaluate the demographic, comorbidities, and hysterectomy type in the sample population by in-hospital mortality. Logistic regression analysis was used to evaluate the demographic and comorbidities that increase the risk of association with in-hospital mortality. A P-value of less than 0.01 was used to determine the statistical significance in all analyses and was conducted using the Statistical Package for the Social Sciences (SPSS), version 26 (IBM Corporation, Armonk, NY).

Ethical approval

Individual identifiers were used to protect the patient's identity and health-related information. So, the use of de-identified NIS database in this study does not require approval from the institutional review board [11].

## Results

We analyzed a total sample of 119,890 inpatients hospitalized for laparoscopic hysterectomy with 17,370 (14.5%) inpatients having comorbid obesity. The majority of the inpatients underwent TLH (51.3%), followed by LAVH (25.8%) and LSH (12.9%).

The majority of the inpatients were middle-age adults 36 to 50 years (83.1%) with obesity cohort older than non-obesity (51.3y vs. 48.8y, P < 0.001). About 67.7% inpatients were White, and when compared to non-obesity, a higher proportion of obese inpatients were black (17.2% vs. 12.7%). Comorbidities were seen in a higher proportion of obesity cohort with most prevalent being hypertension (53.6%) and diabetes (23.9%), followed by depression and hypothyroidism (15.8% and 15.4%, respectively) as shown in Table 1.

**Table 1:** Demographics and hospital outcomes by comorbid obesity in adult inpatients with laparoscopic hysterectomy SD: standard deviation; TLH: total laparoscopic hysterectomy; LAVH: laparoscopic-assisted vaginal hysterectomy; LSH: laparoscopic supracervical hysterectomy

Variable	Laparoscopic hysterectomy		
	Obesity (-)	Obesity (+)	Total
Total inpatients	102,520	17,370	119,890
Mean age, years (SD)	48.8 (12.37)	51.3 (12.42)	-
Age at admission, in %			
18 – 35 years	17.0	16.2	16.9
36 – 50 years	83.0	83.8	83.1
Race, in %			
White	69.2	67.7	68.9
Black	12.7	17.2	13.5
Hispanic	11.1	9.7	10.9
Asian/Pacific Islander	2.4	1.1	2.2
Native American	0.6	0.9	0.6
Others	4.0	3.4	3.9
Comorbidities, in %			
Depression	9.5	15.8	10.6
Diabetes	7.6	23.9	10.4
Hypertension	24.9	53.6	29.9
Hypothyroidism	10.0	15.4	11.0
Hysterectomy type, in %			
LAVH	37.3	28.6	35.8
LSH	13.7	8.9	12.9
TLH	48.9	62.5	51.3
In-hospital mortality, in %	0	0.1	0

**Figure 1:**
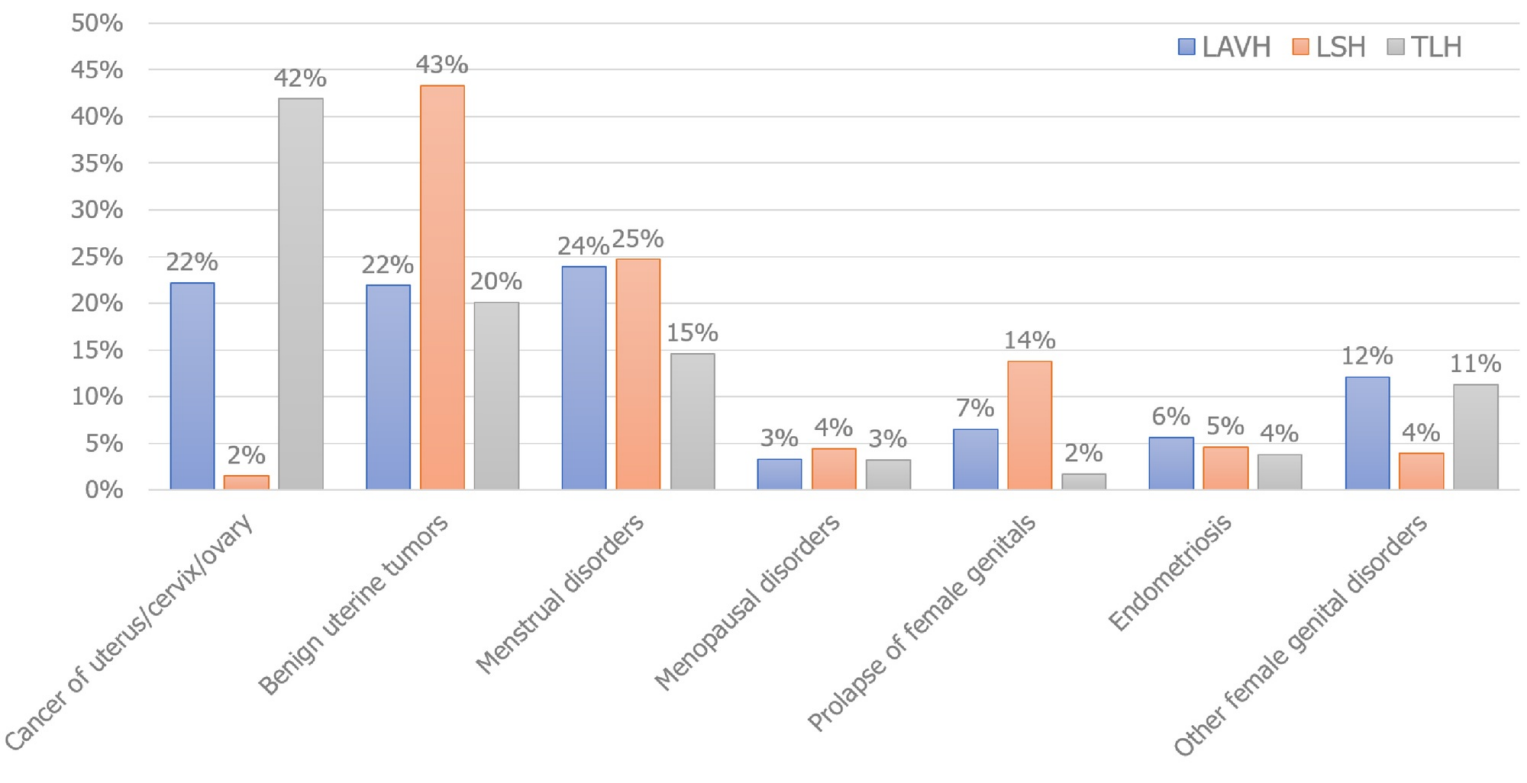
Indications in obese women by hysterectomy type TLH: total laparoscopic hysterectomy; LAVH: laparoscopic-assisted vaginal hysterectomy; LSH: laparoscopic supracervical hysterectomy

When divided by type of hysterectomy, the indications for each type in obese patients varied. The majority of the hysterectomies for TLH were performed for cancer of uterus/cervix/ovary (42%), followed by benign uterine tumors (20%) and menstrual disorders (15%). For LSH, benign uterine tumors were the most common indication accounting for 43% followed by menstrual disorders (25%) and prolapse of female genital organs (14%). LAVH was most commonly performed for menstrual disorders (24%) followed by cancer of uterus/cervix/ovary (22%) and benign uterine tumors (22%) as shown in Figure 1.

Asian/Pacific Islanders and hispanic were at 6.5 times (95% CI 2.51-16.77) and 2.6 times (95% CI 1.29-5.21), respectively, higher risk for in-hospital mortality compared to white. Inpatients with comorbid obesity had 4.6 times (95% CI 2.79-7.69) higher odds for in-hospital mortality compared to non-obesity cohort after controlling for demographic, procedure type, and other comorbidities. There was statistically no significant association between type of laparoscopic hysterectomy and in-hospital mortality as shown in Table 2.

**Table 2:** In-hospital mortality risk in adult inpatients with laparoscopic hysterectomy TLH: total laparoscopic hysterectomy; LAVH: laparoscopic-assisted vaginal hysterectomy; LSH: laparoscopic supracervical hysterectomy

Variable	Logistic regression model			
	Odds ratio	95% confidence interval		P value
		Lower	Upper	
Age at admission	1.12	1.10	1.15	<0.001
Race				
White	Reference			
Black	2.28	1.12	4.65	0.023
Hispanic	2.59	1.29	5.21	0.008
Asian/Pacific Islander	6.49	2.51	16.77	<0.001
Native American	<0.001	<0.001	-	0.990
Others	<0.001	<0.001	-	0.996
Comorbidities				
No comorbidity	Reference			
Depression	4.14	2.41	7.09	<0.001
Diabetes	1.26	0.73	2.19	0.411
Hypertension	0.54	0.31	0.93	0.027
Hypothyroidism	1.11	0.62	1.99	0.737
Obesity	4.64	2.79	7.69	<0.001
Hysterectomy type				
LAVH	1.59	0.96	2.66	0.073
LSH	1.11	0.43	2.86	0.837
TLH	Reference			

**Figure 2:**
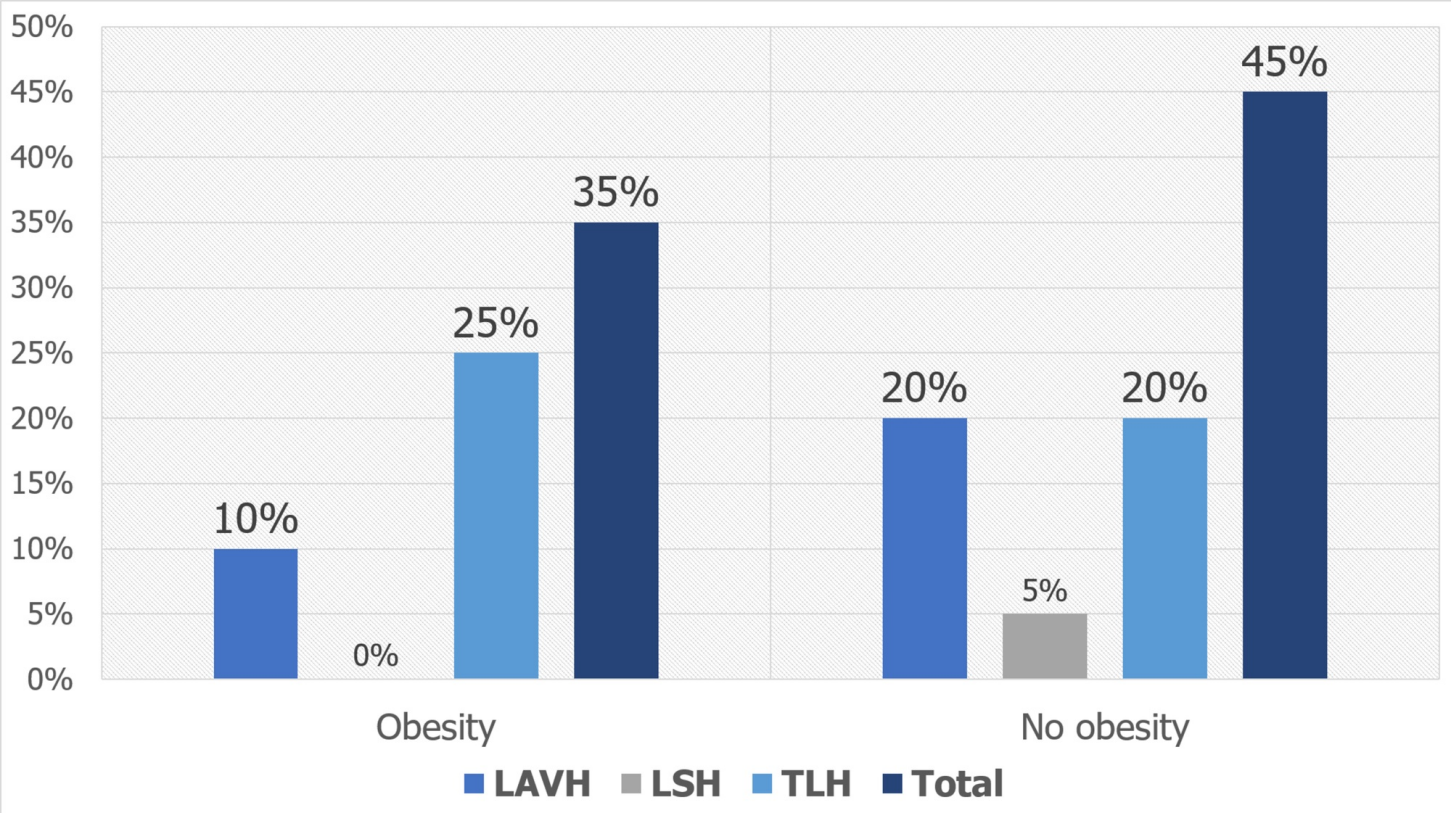
In-hospital mortality by hysterectomy type in adult inpatients TLH: total laparoscopic hysterectomy; LAVH: laparoscopic-assisted vaginal hysterectomy; LSH: laparoscopic supracervical hysterectomy

By proportion, 0.1% (35 out of 17,370) in-hospital mortality was seen in obesity cohort. A higher number of inpatient deaths were reported in patients who underwent TLH (N = 25) and LAVH (N = 10), and none in those undergoing LSH. A total of 45 inpatient deaths were reported in 102,520 inpatients with 20 deaths each in inpatients undergoing TLH and LAVH as shown in Figure 2.

## Discussion

In this study, utilizing the NIS data analysis of 119,890 inpatients hospitalized for primary procedure: laparoscopic hysterectomy, we found that 17,370 (14.5%) had comorbid obesity. When the obese and the non-obese cohorts were compared, we found that there was a higher proportion of comorbidities in the obese cohort with hypertension being the most prevalent, followed by diabetes, depression, and hypothyroidism. Patients with comorbid obesity had a 364% higher risk of in-hospital mortality compared to the non-obese cohort.

More than four-fifth of the inpatients were middle-aged adults (36 to 50 years, 83.1%), with the obesity cohort older than non-obesity. The CDC health trend data also reveals that the prevalence of obesity was highest among middle-aged women and the black population [6]. In our research, 67.7% of total inpatients were white, but when compared to non-obesity, a higher proportion of obese inpatients were black. We found that the Asian/Pacific Islanders and Hispanic populations were at 6.5 times and 2.6 times, respectively, higher risk of in-hospital mortality compared to the White population. This may be because racial minority groups are disproportionately affected by chronic diseases compared to their white counterparts due to lower socioeconomic status and education level, as well as a lack of accessibility to health care and nutritious food. These factors can hinder such groups from receiving adequate medical care and nutrition, leading to an increased incidence of obesity and the risk of complications following surgical procedures [13].

Obesity was deemed to be a relative contraindication for laparoscopy as they are at a higher risk of developing atelectasis as a consequence of the upward displacement of the diaphragm following carbon dioxide insufflation. The loss of muscle tone due to anesthesia and the Trendelenburg position leads to a more definite reduction in functional residual capacity in obese [14-17]. In addition, its association with other comorbid conditions can also negatively impact the outcomes of surgical patients [13,18,19]. In this study, we found that the most common comorbidities were hypertension (53.6%) and diabetes (23.9%). We found a significant association between comorbid obesity and in-hospital mortality in the inpatients for laparoscopic hysterectomy after controlling for demographics and chronic comorbidities. Also, the largeness of the abdominal wall results in difficult access to and visualization of the abdominal cavity, pneumoperitoneum maintenance, and laparoscopic instrument handling [20].

Studies exploring the complication rates in obese and non-obese patients have produced conflicting results. Some have found increased complications and laparo-conversions in obese patients, while other studies suggest that there are no relevant differences between the weight groups [9,10,21]. In a review of 2,530 laparoscopic hysterectomies, those with a BMI higher than 30 kilograms/square meter had a two-fold risk of unintended laparotomy [20,22]. A study by O'Hanlan et al. reported comparable mean operating time, mean operative blood loss, mean length of hospital stay, and complication rates in all BMI groups [18].

While these studies compare the complication rates in the obese and non-obese groups, there is a dearth of literature on the differences in inpatient mortality in at-risk patients undergoing laparoscopic hysterectomy. Therefore, our goal was to examine this outcome comparison because, with the increasing prevalence of obesity, it is of the utmost importance to study, introspect, and implement practices to give a safe outcome to patients. In our study, patients with comorbid obesity had 4.6 times and about 364% higher risk of in-hospital mortality compared to the non-obesity cohort. This was in accordance with a prospective, multi-center study of 118,707 patients who underwent non-bariatric general surgery where higher mortality risk was observed in underweight and morbidly obese [23].

Of the 0.1% (35 out of 17,370) of the in-hospital mortality that was seen in the obesity cohort in our study, a higher number of inpatient deaths were reported in patients who underwent TLH and LAVH, while none were reported in those undergoing LSH. There was no statistically significant association between the type of laparoscopic hysterectomy and in-hospital mortality. Milad et al. reported different findings that showed LAVH to be associated with higher morbidity than LSH [24].

The findings of this study must be seen in the light of some limitations. Firstly, the NIS is an administrative database and not a detailed medical and patient record. We categorized laparoscopic hysterectomy based on ICD-9 procedure codes which are subject to possible miscoding and unable to get details of surgical techniques used. Also, NIS data be deficient in preoperative information: prior abdominal surgeries, size, and descent of the uterus. Next, as ICD-9 diagnostic codes were used to identify comorbid obesity and so it could not be stratified into different classes. Studies have shown that an “obesity paradox” exists, where moderate obesity is protective and mortality is most likely in a morbidly obese individual [25]. However, this phenomenon could not be tested in our study.

## Conclusions

Analysis of national-level data shows that obese patients have a higher risk of in-hospital mortality by 364% compared to non-obese patients. Women with obesity had a higher proportion of chronic comorbidities including hypertension, and diabetes. There was no significant association between the laparoscopy procedure type and in-hospital mortality, yet we found that a higher proportion of inpatient deaths were reported in those who underwent TLH and LAVH. More studies should focus on improving hospital outcomes and quality of life post-laparoscopy in obese women.
